# Cultivated Gut Microbiota of Roe Deer and Red Deer in Central Poland Forest

**DOI:** 10.3390/ani15243656

**Published:** 2025-12-18

**Authors:** Wojciech Ospałek, Łukasz Wlazło, Katarzyna Tajchman, Małgorzata Targońska-Karasek, Bożena Nowakowicz-Dębek

**Affiliations:** 1 Department of Animal Hygiene and Environmental Hazards, University of Life Sciences in Lublin, ul. Akademicka 13, 20-950 Lublin, Poland; wojciech.ospalek@up.edu.pl (W.O.); malgorzata.karasek@up.edu.pl (M.T.-K.); bozena.nowakowicz@up.edu.pl (B.N.-D.); 2 Department of Animal Ethology and Wildlife Management, University of Life Sciences in Lublin, ul. Akademicka 13, 20-950 Lublin, Poland

**Keywords:** intestinal microflora, *Capreolus capreolus*, *Cervus elaphus*

## Abstract

The aim of this study was to compare, using culture methods, the microflora of the small and large intestines, distinguishing pathogenic bacteria, in free-living ruminants: roe deer (*Capreolus capreolus*) and red deer (*Cervus elaphus*). Intestinal samples from six individuals of each species were collected immediately after hunting under aseptic conditions. Aerobic and facultatively anaerobic bacteria, including *Lactobacillus* spp., *Escherichia coli*, *Listeria* spp., and *Clostridium perfringens*, were quantified using standard culture methods. Statistical analysis (ANOVA) revealed no significant differences (*p* > 0.05) between species in any of the microbial groups analyzed, although higher mean abundances were observed in red deer, particularly in the large intestine. The results indicate that interspecific variation in cultured microbiota may reflect individual and environmental factors rather than consistent taxonomic differences. Due to the high inter-individual variability and limited sample size, this study should be considered preliminary. The results demonstrate the predominance of viable aerobic and facultative anaerobic bacterial groups in culture-based analysis and provide reference data for future metagenomic studies. This study fills an important knowledge gap, as culture-dependent studies of the gut microbiota of wild cervids are still rare due to the logistical and ethical constraints associated with sampling wild animals.

## Simple Summary

This study, conducted in Poland, compared the gut microbiota of roe deer (*Capreolus capreolus*) and red deer (*Cervus elaphus*). Samples were collected from the small and large intestines of six individuals of both species during the hunting season. Microbiological analyses revealed interspecies variation in the composition and abundance of microorganisms, although the observed differences were not statistically significant. Red deer had higher numbers of most microorganisms, particularly *Clostridium perfringens*, suggesting differences in gastrointestinal physiology and a high-fiber diet. Roe deer diets are rich in herbs, mushrooms, and cereals, contributing to higher abundances of *E. coli* and *Listeria* spp. in the large intestine. Regardless of species, the large intestine had richer and more diverse microbiota than the small intestine. Seasonal factors, age, and habitat likely influence microbiome composition. Importantly, no Salmonella was detected, indicating a low risk of zoonosis in the study area. These findings highlight the role of the microbiome in digestion, nutritional adaptation, and environmental conditions. Understanding these microbial ecosystems is crucial for monitoring wildlife health, assessing pathogen reservoirs, and assessing environmental impacts. This study complements the limited data on the gut microbiota of native Polish ruminants, with implications for nature conservation and environmental microbiology.

## 1. Introduction

Comprehensive research into intestinal microbiota enables a better understanding of how wild ruminants adapt to changing environmental and seasonal conditions. Microorganisms inhabiting the gastrointestinal tract of these animals play a vital role in digestion and fermentation processes, as well as in maintaining the body’s physiological balance. Both red deer and roe deer intestines are colonized by diverse bacterial communities, with their composition and counts varying depending on the diet, age, season and habitat [1,2,3]. Research results indicate that the microbiome of the roe deer is enriched with characteristic bacterial genera, including *Bifidobacterium, Ruminococcus, Succinivibrio, Treponema, Prevotella* and *Lachnospiraceae* [1]. The digestive tract of the roe deer is also dominated by specific methanogenic archaea, namely *Methanocorpusculum* and *Methanobrevibacter*, but their composition differs from that observed in other ruminants. Interestingly, the predicted activity of metabolic pathways, especially those related to the production of butyrate and propionate, is more intense in roe deer than, for example, in goats, which may affect the rate and efficiency of digestion [1]. In contrast, the intestinal microflora of red deer has a distinctly different structure, primarily dominated by bacteria representing *Firmicutes* and *Proteobacteria* [4]. In these animals, the microbiome also varies with age, as adult individuals have a more developed and diverse flora than younger animals, which may indicate the role of environmental factors in its formation [2,5]. The composition of the microbiota is also significantly affected by seasonal changes. In seasons abundant in fresh and diverse plants (spring-summer), an increase is observed in the abundance and diversity of microorganisms, as compared to the autumn–winter season, when the diet is poorer [6,7]. Similar phenomena have been observed in other deer species, as well as in musk deer, specifically *Moschus berezovskii*, particularly when kept under varying environmental conditions [3]. Free-living ruminants exhibit fluctuations in the numbers of aerobic mesophilic bacteria, fungi, lactobacilli, coliforms, and fecal coliforms, as well as microorganisms pathogenic to humans; the most frequently studied are *Clostridium perfringens*, *Listeria*, and *Salmonella*. Although more is being learned about the functioning of the microbiome in wild animals, there is still limited research on its variability in native species living freely in Poland. Therefore, the objective of the present study was to compare the quantitative composition of main groups of intestinal microorganisms in roe deer (*Capreolus capreolus*) and red deer (*Cervus elaphus*), with particular emphasis on the differences between the small intestine and the large intestine.

## 2. Materials and Methods

### 2.1. An Experimental Project

Material for the study was sourced from six male roe deer (*Capreolus capreolus*) and six male red deer (*Cervus elaphus*), harvested in accordance with Polish hunting laws and the principles of individual selection during the hunting seasons, as part of game animal population management [8]. The animals were harvested in the Lubartów Forest Division area and at the Rawityn and Kozłówka Game Breeding Centre, in central Poland (51°27′ N, 22°29′ E). This region has 24.9% of its area covered by forests, with 49% coniferous forests and 38% mixed forests, including deciduous tree species. The predominant forest type is coniferous forest, where coniferous species make up at least 80% of the total tree species (with Scots pine *Pinus sylvestris* as the dominant species, accounting for 61%). Forests with at least 80% share of deciduous trees (deciduous forests) cover 13% of the area. The most important deciduous tree species include the pedunculate oak (*Quercus robur*) and the sessile oak (*Quercus petraea*)—14%; the silver birch (*Betula pendula*)—8%; the common alder (*Alnus glutinosa*)—6%; the European beech (*Fagus sylvatica*)—3%; and the European hornbeam (*Carpinus betulus*)—2% (https://lubartow.lublin.lasy.gov.pl/, accessed on 26 February 2025). In the Rawityn and Kozłówka Game Breeding Centre area, 321 red deer (*Cervus elaphus*), 104 fallow deer (*Dama dama*), 85 moose (*Alces alces*), and 587 roe deer (*Capreolus capreolus*) were inventoried (unpublished data from the Lubartów Forest Inspectorate, as of 10 March 2023). The individuals under study were harvested in 2023 during the hunting season, i.e., in August for roe deer and in September for red deer, to minimize seasonal differences. The selective culling of the animals was carried out by qualified hunters, employees of the Lubartów Forest Inspectorate. Harvesting the animals involved individual hunts, where particular individuals were approached stealthily from a distance that allowed for a precise shot, without the need for beaters or hunting dogs. The hunts took place in the early morning hours, during quiet feeding times, when the animals were behaving naturally, showing no signs of stress, and were not artificially separated from the herd. Individuals who died immediately after the first precise shot were selected for the study. The age of the animals was determined post-mortem based on their dentition, using Eidmann’s method, which involves the assessment of dentine layers deposited in the canal of the first pair of incisors (I1), with characteristic features indicating the stage of development and continuous replacement of deciduous teeth [9]. The weight of the carcass (without entrails) was determined after the animal was shot and eviscerated at a game animal collection station. As previously mentioned, the culling of game animals was conducted in accordance with Polish hunting laws [8]. Since all hunting activities were conducted as part of hunting management in areas managed by the Lubartów Forest Inspectorate, no additional experimental measures likely to pose a threat to animal welfare were carried out. Therefore, the present study did not require individual consent to be granted by the Local Ethics Committee (Regulations of the Animal Welfare Committee, Faculty of Animal Sciences and Bioeconomy, University of Life Sciences in Lublin, ZdsDz/6/2023).

### 2.2. The Diet of Experimental Animals

*Cervidae* (the deer family) are ruminants whose diet consists mainly of plants found in their habitat. Each species, however, has different dietary preferences. Previous studies have shown that the diet of the red deer was dominated primarily by conifers, dwarf shrubs, and grasses (mainly *Poaceae, Juncaceae* and *Cyperaceae*) (20.3%, 19.0%, and 16.1%, respectively). The diet of the roe deer was dominated by the European dewberry (*Rubus caesius* L., 34.6%), with conifers (14.4%) and dwarf shrubs (10.9%) accounting for a significantly lower percentage. Roe deer consumed more grubs, herbs, wild mushrooms, and cereals than red deer, whose diet consisted mainly of coarse fiber [10].

### 2.3. Sample Collection

The study material came from six male roe deer in August, aged 3–4 years, with an average carcass weight of 19 kg, and six male red deer in September, aged 2–3 years, with an average carcass weight of 127 kg. The small and large intestines were collected from the carcasses during butchering immediately after harvest. The gastrointestinal tract was transferred to sterile containers, cooled during transport (4–6 ± 2 °C), and then transported in thermal bags to the laboratory. The contents of the small and large intestine were collected in sterile containers under laboratory conditions and immediately subjected to microbiological analysis. The microbiological identification procedures were performed according to the methodology described in the Materials and Methods section. To confirm the microbiological identification, biochemical analyses were performed using commercial API tests. The API test procedures were carried out according to the manufacturer’s instructions provided with the kits.

### 2.4. Microbiological Analysis

Samples of the contents of the small and large intestines were analyzed separately for each animal. Ten grams of material were weighed from particular intestinal segments and placed in containers with Ringer’s solution, and a series of dilutions was prepared. Individual dilutions were inoculated onto pre-prepared Petri dishes containing the appropriate culture media. For the analysis of intestinal contents, the following microbial counts were determined using the following microbiological media: 1. total mesophilic aerobic bacterial count on enriched agar (BTL Polska Sp. z o.o., Łódź, Poland); 2. total fungal count on Sabouraud dextrose agar (BTL Polska Sp. z o.o., Łódź, Poland); 3. total coliform bacterial count on Endo les agar (BTL Polska Sp. z o.o., Łódź, Poland); 4. total fecal coliform bacterial count on mFC agar (BTL Polska Sp. z o.o., Łódź, Poland); 5. total *Clostridium perfringens* bacterial count on Tryptose Sulfite Cycloserine Agar (TSC) (Biomerieux Polska Sp. z o.o., Warsaw, Poland); 6. total *Lactobacillus* lactic acid bacterial count on MRS agar (BTL Polska Sp. z o.o., Łódź, Poland); and 7. The presence of *Salmonella* bacilli on XLD agar (BioMerieux Polska Ltd., Warsaw, Poland). The final identification was carried out using API tests (Biomerieux Polska Sp. z o.o., Warsaw, Poland). Each sample was inoculated in triplicate. After the incubation, colonies were counted using a Scan 300 colony counter (Interscience Laboratories, Saint-Nom-la-Bretèche, France), and the counts were expressed as colony-forming units per g of intestinal content [CFU/g].

### 2.5. Statistical Analysis

A statistical analysis was performed to compare the concentration of microorganisms in different sections of the gastrointestinal tract of roe deer and red deer.The results were statistically analyzed using one-way analysis of variance (ANOVA). Levene’s test was used to verify the homogeneity of distribution at *p* < 0.05. The tables contain mean values (M), standard deviation (SD) and the standard error of the mean (SEM). All computations were performed using Statistica software, version 13.3 (StatSoft Inc., Tulsa, OK, USA).

## 3. Results

The small intestine contents of the roe deer were dominated by mesophilic aerobic bacteria, with an average count of 5.2 × 10^6^ CFU/g. A high count was also recorded for lactic acid bacteria of the *Lactobacillus* genus (an average of 3.0 × 10^6^ CFU/g), as well as fecal coliform bacteria (8.8 × 10^5^ CFU/g). The coliform bacterial count was 3.3 × 10^5^ CFU/g. Fungi accounted for a small percentage of the microorganisms determined (an average of 1.2 × 10^4^ CFU/g). The smallest count was shown for *Listeria* (1.5 × 10^3^). *Clostridium perfringens* was only noted in two individuals, whereas the presence of *Salmonella* bacilli was not observed in the roe deer small intestinal samples (Table 1).

An increase in the abundance of most microorganism groups under study was recorded in the large intestine contents of the roe deer compared to the small intestine. The average count of mesophilic aerobic bacteria was 2.2 × 10^8^ CFU/g; for lactic acid bacteria, it was 2.1 × 10^5^ CFU/g. The counts of coliform bacteria and fecal coliform bacteria were correspondingly higher than those in the small intestine (1.5 × 10^6^ CFU/g and 9.0 × 10^5^ CFU/g, respectively). The total fungal count was at a level of 2.1 × 10^5^ CFU/g. Bacteria of the *Listeria* genus were detected in five out of six samples, with an average count of 9.7 × 10^7^. Bacteria of the *Clostridium perfringens* genus were not identified for two individuals, and the average count was 1.5 × 10^4^ CFU/g. In contrast, *Salmonella* was not determined in any of the roe deer large intestine samples (Table 2).

In the red deer, the small intestine contents were dominated by mesophilic aerobic bacteria (an average of 6.6 × 10^7^ CFU/g) and lactic acid bacteria (3.7 × 10^7^ CFU/g). Coliform bacteria and fecal coliform bacterial counts were 1.5 × 10^5^ CFU/g and 6.3 × 10^5^ CFU/g, respectively. The total fungal count determined in the red deer small intestine contents was 2.9 × 10^5^ CFU/g. In the red deer large intestine contents, the counts of all the microorganism groups under study were higher than those in the small intestine. Similar correlations were also noted for roe deer (Table 3). In contrast, in the large intestine of the red deer, a variation in the counts of the particular microorganisms was noted. The average count of mesophilic aerobic bacteria was 6.1 × 10^8^ CFU/g; for lactic acid bacteria, it was 4.0 × 10^8^ CFU/g, and for coliform bacteria, it was 1.0 × 10^5^ CFU/g. Fecal *E. coli* was determined at a level of 1.3 × 10^5^ CFU/g. The total fungal count was at a level of 7.6 × 10^5^ CFU/g. Unlike the roe deer, *Clostridium perfringens* was found in the large intestine of the red deer (an average of 6.0 × 10^3^ CFU/g), whereas no *Salmonella* spp. bacteria were detected in any of the samples of the analyzed material (Table 4).

The smallest differences between the small intestines in the roe deer and the red deer were noted for coliform bacteria (142%) and for *E. coli* bacteria (124%). For the other bacterial group, the result ranged from 170% (mesophilic bacteria and *Lactobacillus*) to 192% (*Listeria* spp.). In the large intestine, the smallest difference was noted for the group *Lactobacillus* (38%), followed by *Clostridium perfringens* (86%), mesophilic bacteria (93%), and fungi (114%). The greatest differences were noted in the large intestine for the populations of *E. coli* (150%), coliform bacteria (175%), and *Listeria* bacilli (177%). The presence of *Clostridium perfringens* was detected in most red deer individuals, in both the small and large intestines. Red deer, on the other hand, showed a higher number of individuals that were not colonized by these bacteria. However, the number of CFUs in the large intestine in particular individuals of roe deer was higher than that of red deer. Red deer tended to show higher average values in most microorganism groups studied in both the small and large intestines, but these differences were not statistically significant (*p* > 0.05). However, in the roe deer, fecal *E. coli* was more abundant in the small and large intestines. A similar trend was observed for bacteria of the coli group and of the *Listeria* genus in the large intestine of the roe deer. Low standard deviations in the large intestine of the two animal groups were observed in mesophilic aerobic bacteria (roe deer—3.5; red deer—1.2), indicating a relatively homogeneous composition of the microbiome in individuals of the same species.

The statistical analysis of microbial concentrations in the small and large intestines of roe deer (*Capreolus capreolus*) and red deer (*Cervus elaphus*) revealed inter-individual variability in microbial counts; however, these differences were not statistically significant. The one-way analysis of variance (ANOVA), performed at a significance level of *p* < 0.05, showed no significant differences between species or intestinal segments for any of the analyzed microbial parameters. The standard error of the mean (SEM) values indicated moderate variability within groups, while the obtained *p*-values (>0.05) confirmed the absence of statistically significant differences in microbial counts between roe deer and red deer (Table 5 and Table 6).

## 4. Discussion

The results revealed significant differences in the intestinal microbiota structure between two species of wild ruminants: roe deer (*Capreolus capreolus*) and red deer (*Cervus elaphus*). The differences were both quantitative (bacterial and fungal counts) and qualitative (presence of specific taxonomic groups) and were clearly visible when comparing microbial counts between the small and large intestines. The data presented in the tables can be associated with dietary differences, environmental conditions, and the physiology of the gastrointestinal tract, highlighting the distinct intestinal ecosystems of the two species [1,7,9,11]. The higher concentrations of all microbial groups found in red deer, along with the more frequent occurrence of *Clostridium perfringens*, suggest differences in digestive physiology and dietary adaptation between species [10,11,12,13]. Red deer, as bulk feeders, consume more fibrous plant material, whereas roe deer, being concentrate selectors, prefer more digestible components such as herbs, shoots, and cereals [7]. Diets rich in crude fiber favor the growth of cellulolytic and fermentative bacteria, particularly in the large intestine, as demonstrated in domestic and wild ruminants [10,11,13]. Similar dietary effects on microbial balance were reported in cattle fed high-grain diets [14], where *E. coli* proliferation and reduced fiber degradation capacity were observed, as well as in humans, where even short-term dietary changes rapidly alter microbial composition [15]. In contrast, roe deer showed lower counts of fiber-degrading bacteria but higher abundance of *E. coli* and *Listeria* spp., which may be linked to their more digestible diet and faster passage rate. The statement referring to the predominance of aerobic mesophilic bacteria relates exclusively to the results obtained under aerobic culture conditions. This does not imply that these microorganisms are dominant in the total intestinal microbiota. The applied methods allowed only for the detection and quantification of viable, cultivable fractions of the gut microbiota that can grow in aerobic or facultatively anaerobic conditions. The absence of *Clostridium perfringens* in roe deer and its presence in red deer suggest physiological differences, such as feed retention time, pH, and availability of branched-chain amino acids [11,12,13]. Species with longer digesta retention are more likely to support proteolytic anaerobic bacteria, including potentially pathogenic *Clostridium* spp. [4,16]. These interspecies differences in fermentation efficiency are consistent with the findings of Han et al. [1], who showed that lower methane production in roe deer correlates with reduced activity of fiber-degrading bacteria. The relationship between diet, fermentation, and microbial activity supports the conclusion that microbial structure directly reflects ecological and nutritional specialization. An important factor shaping the microbiome is age. Previous studies have shown that microbial diversity stabilizes and increases with age [2,5,10], which may have been relevant here since the roe deer were slightly older than the red deer. Age classification in this study was standardized based on the molar wear scoring method of Brown and Chapman [17], ensuring consistency among individuals. The observed differences between small and large intestines within each species are consistent with previous reports. The large intestine, being the main site of anaerobic fermentation, contained higher microbial counts, including fermentative bacteria and enterobacteria, while fungi were less numerous due to competition with bacteria and sensitivity to environmental fluctuations [13,18,19,20]. These findings are in line with Li et al. [6] and Cui et al. [13], who observed similar microbial gradients in red deer and other wild ruminants. Comparative research on captive and free-ranging animals [21] further indicates that environmental conditions and diet stability are crucial for maintaining microbial diversity. Seasonal and environmental variation also appear to be important determinants of microbial composition. In this study, samples from roe deer were collected in summer and from red deer in autumn, which may partly explain the observed differences [5,6,22,23]. Seasonal shifts in feed composition are known to cause microbial adjustments; for example, Amato et al. [24] observed compensatory microbial changes in wild primates, and similar mechanisms likely occur in wild ruminants. Guo et al. [23] confirmed that both environmental and dietary gradients significantly influence microbial diversity in wild deer populations. Pathogenic bacteria relevant to humans were also identified. *E. coli* and *Listeria* spp. were more frequent in roe deer, especially in the large intestine, whereas *Salmonella* spp. were not detected in any sample, confirming low zoonotic risk and favorable hygiene [16,18,25]. Szczerba-Turek et al. [12] reported that nearly 25% of wild ruminants carry Shiga toxin–producing *E. coli* (STEC). These findings highlight that wild ungulates can serve as emerging reservoirs for zoonotic pathogens, especially as anthropogenic pressure increases [12,14]. Moreover, studies of free-ranging roe deer in agricultural habitats [22] show that environmental stress may alter microbial balance and increase susceptibility to opportunistic species, emphasizing the ecological importance of microbiota monitoring. In summary, the intestinal microbiota of roe and red deer represents a dynamic, adaptive ecosystem influenced by diet, season, age, and environmental conditions. The large intestine consistently harbors more abundant and diverse microbial communities than the small intestine in both species. The inclusion of physiological, ecological, and environmental perspectives in this study supports the view that microbial diversity acts as an indicator of animal health and ecosystem balance. Intestinal material from wild animals is difficult to obtain and standardize (due to time from shooting to collection, environmental conditions, and seasonality), which limits sample size and the range of methods. The rarity of culture-dependent studies in this taxonomic group supports the validity of reporting preliminary data and using them as a starting point for further analyses using larger samples and sequencing methods. Although no statistically significant interspecies differences were found (*p* > 0.05), clear trends were observed, indicating higher mean microbial counts in red deer and greater individual variability in roe deer. These phenomena may reflect differences in diet composition, metabolic activity levels, and gastrointestinal adaptation. The results confirm the overall similarity of the cultured microbiota of both species, while the existence of biological qualitative differences requiring further verification. The authors would like to emphasize that this study is preliminary and includes a limited number of samples. This, combined with the high individual variability typical of wild species, limits the possibility of obtaining statistically significant differences. The methodology used, based on the cultivation of aerobic and facultatively anaerobic microorganisms, allowed for the assessment of viable microbiome fractions but did not encompass the full spectrum of anaerobic cultures, with the exception of *Clostridium perfringens*, which limits the presented research. An additional limitation is the difficulty in obtaining and standardizing intestinal material from wild animals (due to time from shooting to collection, seasonality, and environmental conditions). Despite these limitations, the obtained results provide a valuable reference point for future metagenomic studies on larger numbers of individuals.

## 5. Conclusions

The study indicates interspecies variability in the composition and abundance of the gut microbiota in roe deer and red deer. Although the observed tendencies (higher counts in red deer, especially in the large intestine) were not statistically significant, they may reflect physiological and dietary adaptations. The roe deer microbiota, however, showed diversity within individual bacterial generation, with lower overall abundance and more intense activity of some fermentation pathways. Differences also occurred in the presence of *Clostridium perfringens*, as this bacterium was found primarily in red deer. *Salmonella* spp. did not grow colonies on plates in samples from either species, indicating favorable habitat conditions. Seasonal variation, dietary diversity, age, and habitat specificity played a key role in shaping the gut microbiome ecosystem. These findings demonstrate the complex interrelationships between the environment, animals, their diet, and their bacterial symbionts. A deeper understanding of microbial dynamics in wild ruminants enables the development of conservation strategies, promoting wildlife health, and contributing to broader ecological knowledge, including aspects related to nutrient cycling in forest ecosystems.

In recent years, it has been noted that the microbiome profile of cervids, particularly the European roe deer (*Capreolus capreolus*), is poorly understood—for example, the first metagenomic study of the fecal microbiome of this species was only recently published [26]. Our study fills this gap by providing data on viable microbiota fractions, including taxa of sanitary importance (*E. coli*, *Listeria* spp., *Clostridium perfringens*). These data can serve as a reference for future metagenomic studies conducted on larger numbers of individuals and across different seasons. Due to the limited number of studies on the composition and variability of the gut microbiota of wild ruminants, this study contributes to the development of knowledge about their digestive physiology and interactions with the environment. The obtained results confirmed the research hypothesis that both species and habitat influence the composition of the gut microbiota, therefore, further analyses using next-generation sequencing techniques are warranted to reflect the qualitative and quantitative composition of the gut.

## Figures and Tables

**Table 1:** Microbial counts in the small intestine contents of roe deer (CFU/g).

	Small Intestine of Roe Deer/Individual Number							
Parameters Tested	1	2	3	4	5	6	M	SD
Total mesophilic aerobic bacterial count	2.5 × 10^6^	2.3 × 10^7^	1.3 × 10^6^	3.7 × 10^3^	2.3 × 10^6^	2.1 × 10^6^	5.2 × 10^6^	8.8 × 10^1^
Total fungal count	>1.0 × 10^1^	1.0 × 10^3^	5.0 × 10^3^	>1.0 × 10^1^	1.0 × 10^3^	6.2 × 10^4^	1.2 × 10^4^	2.5 × 10^4^
Total *Lactobacillius* lactic acid bacterial count	2.4 × 10^6^	1.3 × 10^7^	6.5 × 10^4^	>1.0 × 10^1^	5.7 × 10^4^	2.5 × 10^6^	3.0 × 10^6^	5.0 × 10^6^
Total coliform bacterial count	7.4 × 10^3^	1.3 × 10^5^	3.0 × 10^5^	1.0 × 10^3^	4.8 × 10^6^	2.9 × 10^4^	8.8 × 10^5^	1.9 × 10^6^
Total fecal coliform bacterial count	1.3 × 10^3^	1.7 × 10^4^	6.6 × 10^5^	5.0 × 10^2^	9.0 × 10^2^	2.0 × 10^5^	1.5 × 10^5^	2.6 × 10^5^
*Clostridium perfringens* bacterial count	1.1 × 10^3^	4.0 × 10^2^	neg.	neg.	neg.	neg.	2.5 × 10^2^	4.5 × 10^2^
Total *Listeria* bacterial count	4.4 × 10^3^	4.0 × 10^2^	neg.	3.0 × 10^2^	4.1 × 10^3^	neg.	1.5 × 10^3^	2.1 × 10^3^
Presence of *Salmonella* bacilli	neg.	neg.	neg.	neg.	neg.	neg.	-	-

neg.—not found in the analyzed material.

**Table 2:** Microbial counts in the large intestine contents of roe deer (CFU/g).

	Large Intestine of Roe Deer/Individual Number							
Parameters Tested	1	2	3	4	5	6	M	SD
Total mesophilic aerobic bacterial count	1.5 × 10^4^	1.2 × 10^6^	9.6 × 10^5^	1.5 × 10^5^	7.6 × 10^8^	5.7 × 10^8^	2.2 × 10^8^	3.5 × 10^1^
Total fungal count	>1.0 × 10^1^	1.5 × 10^3^	3.5 × 10^4^	>1.0 × 10^1^	1.0 × 10^3^	1.2 × 10^6^	2.1 × 10^5^	4.9 × 10^5^
Total *Lactobacillius* lactic acid bacterial count	3.8 × 10^2^	4.7 × 10^5^	2.2 × 10^5^	1.1 × 10^5^	1.1 × 10^9^	5.6 × 10^8^	2.8 × 10^8^	4.6 × 10^8^
Total coliform bacterial count	7.2 × 10^4^	6.5 × 10^5^	7.4 × 10^5^	2.4 × 10^4^	1.0 × 10^6^	6.7 × 10^6^	1.5 × 10^6^	2.6 × 10^6^
Total fecal coliform bacterial count	6.6 × 10^4^	5.7 × 10^5^	2.7 × 10^5^	2.6 × 10^4^	5.4 × 10^4^	4.4 × 10^6^	9.0 × 10^5^	1.7 × 10^6^
*Clostridium perfringens* bacterial count	4.8 × 10^3^	neg.	3.1 × 10^4^	2.4 × 10^4^	1.3 × 10^4^	neg.	1.5 × 10^4^	1.5 × 10^4^
Total *Listeria* bacterial count	1.5 × 10^3^	neg.	1.1 × 10^4^	2.3 × 10^3^	4.6 × 10^3^	3.9 × 10^4^	9.7 × 10^3^	1.5 × 10^4^
Presence of *Salmonella* bacilli	neg.	neg.	neg.	neg.	neg.	neg.	-	-

neg.—not found in the analyzed material.

**Table 3:** Microbial counts in the small intestine contents of red deer (CFU/g).

	Small Intestine of Red Deer/Individual Number							
Parameters Tested	1	2	3	4	5	6	M	SD
Total mesophilic aerobic bacterial count	1.5 × 10^5^	2.5 × 10^7^	1.0 × 10^8^	3.7 × 10^5^	2.5 × 10^8^	2.1 × 10^7^	6.6 × 10^7^	9.6 × 10^1^
Total fungal count	1.5 × 10^6^	3.5 × 10^4^	1.8 × 10^5^	4.6 × 10^4^	4.2 × 10^3^	>1.0 × 10^1^	2.9 × 10^5^	5.9 × 10^5^
Total *Lactobacillius* lactic acid bacterial count	1.6 × 10^6^	2.0 × 10^7^	4.6 × 10^7^	1.3 × 10^5^	1.3 × 10^8^	2.5 × 10^7^	3.7 × 10^7^	4.9 × 10^7^
Total coliform bacterial count	4.0 × 10^3^	1.9 × 10^5^	2.7 × 10^5^	1.7 × 10^5^	1.8 × 10^5^	8.7 × 10^4^	1.5 × 10^5^	9.2 × 10^4^
Total fecal coliform bacterial count	1.8 × 10^4^	1.2 × 10^6^	3.5 × 10^5^	2.6 × 10^5^	1.8 × 10^6^	1.3 × 10^5^	6.3 × 10^5^	1.7 × 10^5^
*Clostridium perfringens* bacterial count	neg.	4.0 × 10^3^	neg.	2.2 × 10^4^	>1.0 × 10^1^	>1.0 × 10^1^	4.3 × 10^3^	8.8 × 10^3^
Total *Listeria* bacterial count	3.3 × 10^3^	1.4 × 10^4^	4.3 × 10^4^	neg.	3.9 × 10^5^	neg.	7.5 × 10^4^	1.6 × 10^5^
Presence of *Salmonella* bacilli	neg.	neg.	neg.	neg.	neg.	neg.	-	-

neg.—not found in the analyzed material.

**Table 4:** Microbial counts in the large intestine contents of red deer (CFU/g).

	Large Intestine of Red Deer/Individual Number							
Parameters Tested	1	2	3	4	5	6	M	SD
Total mesophilic aerobic bacterial count	8.6 × 10^5^	1.4 × 10^6^	2.1 × 10^8^	3.1 × 10^6^	3.0 × 10^9^	4.4 × 10^8^	6.1 × 10^8^	1.2 × 10^1^
Total fungal count	4.2 × 10^6^	5.4 × 10^3^	1.6 × 10^4^	2.6 × 10^5^	3.8 × 10^4^	1.2 × 10^4^	7.6 × 10^5^	1.7 × 10^6^
Total *Lactobacillius* lactic acid bacterial count	1.2 × 10^7^	3.2 × 10^6^	1.3 × 10^8^	1.3 × 10^6^	1.9 × 10^9^	3.8 × 10^8^	4.0 × 10^8^	7.5 × 10^7^
Total coliform bacterial count	5.4 × 10^4^	6.3 × 10^4^	1.4 × 10^4^	3.1 × 10^5^	3.3 × 10^3^	1.7 × 10^5^	1.0 × 10^5^	1.2 × 10^5^
Total fecal coliform bacterial count	2.4 × 10^4^	5.4 × 10^4^	1.4 × 10^4^	2.4 × 10^5^	2.4 × 10^5^	1.9 × 10^5^	1.3 × 10^5^	1.1 × 10^5^
*Clostridium perfringens* bacterial count	2.5 × 10^4^	1.1 × 10^4^	>1.0 × 10^1^	>1.0 × 10^1^	>1.0 × 10^1^	>1.0 × 10^1^	6.0 × 10^3^	1.0 × 10^4^
Total *Listeria* bacterial count	3.7 × 10^4^	neg.	1.1 × 10^5^	neg.	1.9 × 10^5^	6.3 × 10^5^	1.6 × 10^5^	2.4 × 10^5^
Presence of *Salmonella* bacilli	neg.	neg.	neg.	neg.	neg.	neg.	-	-

neg.—not found in the analyzed material.

**Table 5:** Comparison of microorganism concentrations in the small intestine of roe deer (*Capreolus capreolus*) and red deer (*Cervus elaphus*) (CFU/g).

Parameters Tested	Roe Deer (*Capreolus capreolus*)	Red Deer (*Cervus elaphus*)	SEM	*p*
Total mesophilic aerobic bacterial count	5.20 × 10^6^	6.58 × 10^7^	2.11 × 10^7^	0.161
Total fungal count	1.15 × 10^4^	2.94 × 10^5^	1.23 × 10^5^	0.271
Total *Lactobacillius* lactic acid bacterial count	3.00 × 10^6^	3.71 × 10^7^	1.08 × 10^7^	0.118
Total coliform bacterial count	8.78 × 10^5^	1.50 × 10^5^	3.91 × 10^5^	0.377
Total fecal coliform bacterial count	1.47 × 10^5^	6.26 × 10^5^	1.64 × 10^5^	0.153
*Clostridium perfringens* bacterial count	2.50 × 10^2^	4.34 × 10^3^	1.82 × 10^3^	0.283
Total *Listeria* bacterial count	1.53 × 10^3^	7.50 × 10^4^	3.22 × 10^4^	0.273

Data are presented as mean values (M) ± standard error of the mean (SEM). Differences were considered statistically significant at *p* < 0.05.

**Table 6:** Comparison of microorganism concentrations in the large intestine of roe deer (*Capreolus capreolus*) and red deer (*Cervus elaphus*) (CFU/g).

Microorganism Group	Roe Deer (*Capreolus capreolus*)	Red Deer (*Cervus elaphus*)	SEM	*p*
Total mesophilic aerobic bacterial count	2.22 × 10^8^	5.23 × 10^8^	2.30 × 10^8^	0.538
Total fungal count	2.06 × 10^5^	6.84 × 10^5^	3.24 × 10^5^	0.487
Total *Lactobacillius* lactic acid bacterial count	2.77 × 10^8^	3.47 × 10^8^	1.60 × 10^8^	0.838
Total coliform bacterial count	1.53 × 10^6^	1.32 × 10^5^	5.01 × 10^5^	0.174
Total fecal coliform bacterial count	8.98 × 10^5^	1.43 × 10^5^	3.29 × 10^5^	0.270
*Clostridium perfringens* bacterial count	1.51 × 10^4^	5.15 × 10^3^	3.61 × 10^3^	0.178
Total *Listeria* bacterial count	9.73 × 10^3^	1.38 × 10^5^	4.85 × 10^4^	0.199

Data are presented as mean values (M) ± standard error of the mean (SEM). Differences were considered statistically significant at *p* < 0.05.

## Data Availability

The data presented in this study are available on request from the corresponding author.
